# A Review of the First 100 Cases of Laparoscopic Nephrectomy: A Single-Center Experience

**DOI:** 10.7759/cureus.20964

**Published:** 2022-01-05

**Authors:** Muhammad Shoaib Mithani, Waqar Hassan, Muhammad Hammad Ali Mithani

**Affiliations:** 1 Urology, The Kidney Centre Postgraduate Training Institute, Karachi, PAK; 2 Urology, Dow University of Health Sciences, Dow International Medical College, Karachi, PAK

**Keywords:** medical technology, early discharge, nephrectomy, learning curve, laparoscopy

## Abstract

Objective

Since 1990, when the first laparoscopic nephrectomy was performed, there has been a dramatic increase in interest in laparoscopic procedures in urological surgeries.The aim of the study was to present our experience with the first 100 cases of laparoscopic nephrectomy at our institute, identify the difficulties encountered, and discuss how to approach the solutions.

Materials and methods

The data of all patients who underwent laparoscopic nephrectomy between May 2017 and April 2019 (n=100) were reviewed retrospectively.

Results

A total of 100 patients (49 men and 51 women), with a mean age of 34.1 ± 15.1 years, underwent laparoscopic nephrectomy. The mean operative time was 108 min (IQR, 45-240). The operative time was significantly reduced with the surgeons' experience. Of 100 cases, only four were converted to open surgery because of bleeding from the renal bed area, strong adhesions from previous surgeries, or morbid obesity. Of 100 patients, 30 were allowed intake on the same operative day while 70 were allowed on the first postoperative day. Intravenous and oral analgesics were discontinued on the second postoperative day in 81 patients.

Conclusion

The learning curve in our series of cases is comparable to a very similar studywith reduced operative time, reduced blood loss, and fewer complications when compared to open nephrectomy. In addition, setups with minimal previous laparoscopic units can initiate laparoscopic procedures with minimal risk to the patient.

## Introduction

In 1990, the first laparoscopic nephrectomy was performed on an elderly patient for a 3-cm renal mass by Clayman et al. [1]. Since then, the interest in laparoscopic nephrectomy has increased, and many urologists started performing laparoscopic nephrectomy. Being particularly easier to master, the laparoscopic approach in nephrectomy has become the most common laparoscopic surgery in urology [2]. Since then, many studies have been presented by several urologists who performed laparoscopic surgeries and compared the open approach with other laparoscopic approaches. Laparoscopic nephrectomy was proven to be better than open nephrectomy because of the lesser need for postoperative analgesics, faster return to normal activities, lower morbidity rate, and potentially lower costs [3-7]. However, there is a significant learning curve associated with laparoscopic nephrectomy in addition to the establishment of the setup, training of well-trained technicians, and care and proper use of instruments [8].

With the world moving toward the laparoscopic approach, our center provided an opportunity to start laparoscopic nephrectomy.

This study aimed to present our experience with the first 100 cases of laparoscopic nephrectomy at our institution, all performed by one surgeon. Well-established laparoscopic centers are already performing laparoscopic urological procedures. We wanted to determine if it was possible to start laparoscopy at our center where there was no previous established setup and how long it would take before we perform more than 100 laparoscopic surgeries. The secondary objective was to identify the difficulties encountered and discuss how to approach the solutions.

## Materials and methods

This study was conducted at The Kidney Centre Postgraduate Training Institute, Karachi, between May 2017 and April 2019. The Kidney Centre Ethical Review Committee approved the study. Data of all patients who underwent laparoscopic nephrectomy between May 2017 and April 2019 (n = 100) were reviewed retrospectively. The data were collected from electronic medical records after acquiring approval from the ethical review committee. The data were stored in electronic form on the primary investigator’s laptop. The data were collected retrospectively from the clinical records and reviewed (i.e., age, sex, patient’s weight, kidney size, side of surgery, indication for surgery, operative time, postoperative analgesia requirement, blood loss, hospital stay, and need for blood transfusion). Data collection was conducted by a primary investigator, and the patient’s personal details were not recorded to ensure anonymity. Data were entered and analyzed in IBM SPSS version 20 (IBM Corp. Armonk, NY). Cleaning and coding of data were performed before the analysis. Mean ± standard deviation (SD) or median with interquartile range (IQR) was computed for continuous data, whereas frequency with percentages was obtained for categorical data. The normality of data was checked by Shapiro-Wilk tests.

Surgical procedure for nephrectomy

These laparoscopic surgeries were performed by a single surgeon. The transperitoneal approach was used in laparoscopic nephrectomy. The patient was placed in the flank position. Three trocars were used for the procedure. The first trocar (10 mm) was placed lateral to the rectus sheath at the umbilical level. The pneumoperitoneum was achieved using the open Hasson technique [9]. The second and third trocars (both 5 mm) were placed at the anterior axillary line in such a way that the triangle was made.

The colon was reflected medially until Gerota’s fat and the psoas muscle were identified. Along the psoas muscle, the ureter was identified and lifted in a sling with the help of a needle placed under vision at the mid-axillary line. The ureter was followed proximally; the kidney was mobilized up to the hilum. The ureter was clipped and incised first to help mobilize the kidney from the posterior side. Metal clips were used to fasten the renal artery and vein before dividing. Then, the specimen was retrieved with the help of an organ retrieval bag.

## Results

We enrolled 100 patients in our study (49 men and 51 women). The mean age was 34.1 ± 15.1 years, with a minimum age of 12 years and a maximum age of 72 years. Other demographic and clinical variables are shown in Table 1.

**Table 1:** Demographics and clinical parameters of patients

Variables	Mean±std	Minimum	Maximum
Age (years)	34.1±15.1 years	12 years	72 years
Weight (kg)	59.1± 13.9 kg	34 kg	95 kg
Kidney size (cm)	9.1± 3.2 cm	2 cm	17 cm
Blood loss (ml)	50±70 ml	10 ml	600 ml
Hospital stay duration (days)	3.5±1.75 days	2 days	7 days

Of the 100 patients, 54 (54%) underwent right-sided nephrectomy and 46 (46%) underwent left-sided nephrectomy. A total of 32 patients (32%) had a history of abdominal surgery, i.e., appendectomy, C-section, pyelolithotomy, and inguinal hernia repair. Of 100 patients, 25 (25%) had a nonfunctioning atrophic kidney, 41 (41%) had a nonfunctioning dilated kidney, and 34 (34%) had pyonephrotic kidneys. The mean operative time was 108 min (IQR, 45-240). Of 100 patients, 66% had a total operative time of <60 min. The mean operative time was reduced in subsequent cases with gain in expertise over time.

Very few complications were observed in this technique of surgery, as only one patient had wound infection and one patient had prolonged ileus, while only two patients needed a blood transfusion. Of the 100 patients who underwent laparoscopic surgeries, only four were converted to open surgery. In two patients, there was uncontrolled bleeding from the renal bed area, and in one patient, there was a failure to progress due to strong adhesions from previous surgeries. Moreover, one patient had difficulty in port placement because of morbid obesity.

**Figure 1:**
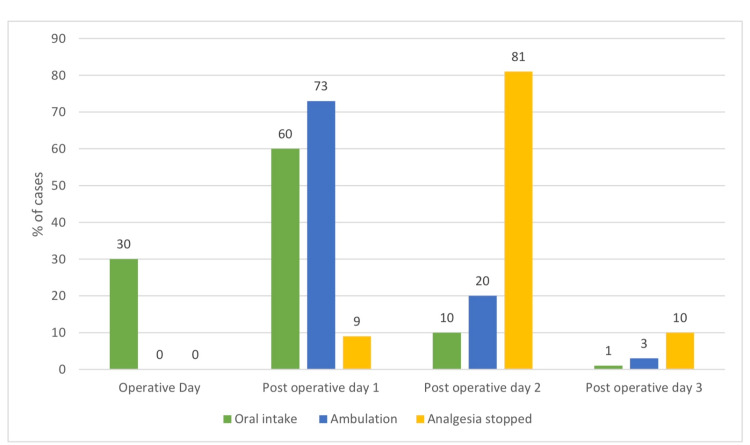
Postoperative parameters

Thirty patients were allowed oral intake on the same operative day, and 60 patients on the first postoperative day (Figure 1). Moreover, 73 patients were mobilized on the first postoperative day. Intravenous and oral analgesics were discontinued on the second postoperative day in 81 patients (Figure 1).

## Discussion

A total of 100 patients underwent laparoscopic nephrectomy. Based on the surgeon’s preference, the approach was transperitoneal in all cases. From our results, it is evident that, although there is a learning curve in the transperitoneal approach for nephrectomy, even relatively new centers can establish their laparoscopy unit with minimal consequences if it is being established by a responsible team. Our results show that even a relatively new center (in terms of laparoscopic expertise) can start their laparoscopic urological procedures without significant morbidity and mortality. As the learning curve for laparoscopy is poorly defined, establishing a laparoscopic setup in a framework with limited to no previous laparoscopic experience was a challenge. Our experience of starting a laparoscopy unit from scratch and achieving 100 laparoscopic nephrectomies demonstrates results that are comparable to the studies from other centers [10].

Patients had a median age of 31 (12-72) years and a median weight of 59 (34-95) kg. The male-to-female ratio was 49:51. Moreover, 38 patients had a history of abdominal surgery, including pyelolithotomy, exploratory laparotomy, C-section, and appendectomy. Performing laparoscopic surgery in a patient who has a history of surgery in the same region is technically difficult; therefore, of the four patients who underwent procedures that were converted to open surgery, two had previous abdominal surgery, and there was a failure to progress. It is also evident from the literature that laparoscopic surgery in a previously operated case is difficult and there are higher risks of conversion to open surgery but it does not lead to a higher rate of complications [11].

There was an exponential increase in the number of cases in two years, which reflects increased expertise, confidence, and the unit’s development. In the first six months, we managed to perform laparoscopic nephrectomy of only three patients, while in the last six months, there were 53 patients.

The mean operative time was 108 min (IQR, 45-240). Of 100 patients, 66% had a total operative time of <60 min. The mean operative time was reduced in subsequent cases with the gain in expertise over time. With this result, we concluded that operative time is a good marker that reflects the learning curve of a surgeon.

Although the blood loss was <50 mL in 60% of cases, the mean blood loss was 86 mL. As the blood loss was between 200 and 250 mL in the initial few cases, the mean blood loss increased.

Several studies comparing open nephrectomy and laparoscopic nephrectomy have been published and found that the laparoscopic approach is better than the open approach because of shortened postoperative hospital stay, lesser blood loss, and decreased analgesic requirement, with no significant difference in complication rate [5].

All laparoscopic nephrectomies were completed successfully, except in four cases, which required conversion to open surgery. There was uncontrolled bleeding from the renal bed area in two cases, failure to progress in one case because of strong adhesions from the previous surgeries, and difficulty in port placement in one case because of morbid obesity.

Of the 100 patients, 45 were discharged on the first postoperative day, indicating a hospital stay of two days. This is either equal to or even less than the length of stay from some previously published series. Early discharge indicated that the bed was available for the next patient who was in need of medical care. In a country like ours, where medical care is a luxury and the number of hospital beds per 1000 people is only 0.6 (data from the World Bank [12]), early recovery and availability of beds are crucial.

This study included only the first 100 cases of laparoscopic nephrectomy. The outcome could have been better predicted with a larger-scale study. The outcome may vary depending on the surgical experiences and the patient’s physical condition.

## Conclusions

From our study, we conclude that the learning curve in our series of cases is comparable to a very similar study with shorter operative time, less blood loss, and fewer complications compared to open nephrectomy. Moreover, a setup with no previous laparoscopic units can initiate laparoscopic procedures with minimal risk to the patient. The benefits of laparoscopic procedures are numerous, and patients should not be denied such benefits due to lack of availability.

## References

[1] Clayman RV, Kavoussi LR, Soper NJ (1991). Laparoscopic nephrectomy: initial case report. J Urol.

[2] Siqueira JR TM, Kuo RL, Gardner TA (2002). Major complications in 213 laparoscopic nephrectomy cases: the Indianapolis experience. J Urol.

[3] Kerbl K, Clayman RV, McDougall EM (1994). Transperitoneal nephrectomy for benign disease of the kidney: a comparison of laparoscopic and open surgical techniques. Urology.

[4] Wilson BG, Deans GT, Kelly J, McCrory D (1995). Laparoscopic nephrectomy: initial experience and cost implications. Br J Urol.

[5] Fornara P, Doehn C, Friedrich HJ, Jocham D (2001). Nonrandomized comparison of open flank versus laparoscopic nephrectomy in 249 patients with benign renal disease. Eur Urol.

[6] Gill IS, Clayman RV, McDougall EM (1995). Advances in urological laparoscopy. J Urol.

[7] Meraney AM, Gill IS (2002). Financial analysis of open versus laparoscopic radical nephrectomy and nephroureterectomy. J Urol.

[8] Higashihara E, Baba S, Nakagawa K (1998). Learning curve and conversion to open surgery in cases of laparoscopic adrenalectomy and nephrectomy. J Urol.

[9] Hasson HM (1971). A modified instrument and method for laparoscopy. Am J Obstet Gynecol.

[10] Phillips J, Catto JW, Lavin V, Doyle D, Smith DJ, Hastie KJ, Oakley NE (2005). The laparoscopic nephrectomy learning curve: a single centre's development of a de novo practice. Postgrad Med J.

[11] Freys SM, Fuchs KH, Heimbucher J, Thiede A (1994). Laparoscopic interventions in previously operated patients [Article in German]. Chirurg.

[12] (2020). The World Bank, Pakistan data. https://data.worldbank.org/country/pakistan.

